# Foundational basic animal experiments paved the way for the clinical development of uterus transplantation

**DOI:** 10.1002/rmb2.12656

**Published:** 2025-05-21

**Authors:** Iori Kisu, Makoto Mihara, Hisako Hara, Yojiro Kato, Yusuke Matoba, Yohei Yamada, Kentaro Matsubara, Hideaki Obara, Nobuhiko Suganuma, Kouji Banno

**Affiliations:** ^1^ Department of Obstetrics and Gynecology Keio University School of Medicine Tokyo Japan; ^2^ Lymphedema Clinic Tokyo Tokyo Japan; ^3^ Department of Lymphatic and Reconstructive Surgery JR Tokyo General Hospital Tokyo Japan; ^4^ Division of Gastroenterological & General Surgery, Department of Surgery, School of Medicine Showa University Tokyo Japan; ^5^ Department of Obstetrics and Gynecology Hiroshima University Hospital Hiroshima Japan; ^6^ Department of Pediatric Surgery Keio University School of Medicine Tokyo Japan; ^7^ Setagaya Vascular Clinic Tokyo Japan; ^8^ Department of Surgery Keio University School of Medicine Tokyo Japan; ^9^ Department of Obstetrics and Gynecology, Bantane Hospital Fujita Health University School of Medicine Nagoya Japan; ^10^ Center of Maternal‐Fetal/Neonatal Medicine Hiroshima University Hospital Hiroshima Japan

**Keywords:** animal research, clinical application, non‐human primate, uterine factor infertility, uterus transplantation

## Abstract

Uterus transplantation (UTx) has emerged as a revolutionary treatment for absolute uterine factor infertility, made possible by extensive preclinical research. Animal studies have played a pivotal role in advancing UTx to clinical practice. We highlight Japan's contribution, including non‐human primate research and regulatory progress, leading to its anticipated clinical implementation. The expansion of UTx in the Asia‐Oceania region underscores its global impact. Further refinements in surgical techniques, optimization of immunosuppressive regimens, and establishment of clear patient eligibility criteria will be crucial for ensuring the long‐term success and sustainability of UTx programs worldwide. This letter acknowledges the Swedish team's foundational efforts in establishing UTx as a viable reproductive technology.

Dear Editor,

We read with great interest the recent review by Brännström et al., which provides a comprehensive overview of the transition from preclinical research to clinical applications in uterus transplantation (UTx).^1^ We fully concur with their statement that “animal studies have been pivotal in transitioning uterus transplantation into clinical practice.” We appreciate their recognition of the extensive foundational research in Japan that has contributed to the advancement of UTx.^2^, ^3^

Brännström et al. pioneered over a decade of foundational animal research, culminating in the first successful live birth following UTx in Sweden in 2014.^4^ This milestone paved the way for the global expansion of UTx. Currently, more than 140 UTx procedures have been performed worldwide, resulting in over 70 live births, and more than 20 medical teams are now engaged in this transformative field of reproductive medicine.^1^

Japan has significantly contributed to UTx research, beginning in 2009 with non‐human primate models.^2^, ^3^ Inspired by the Swedish team's research in baboons,^5^ we initiated studies using cynomolgus macaques to refine surgical techniques and immunosuppressive strategies. Our achievement of live births following autologous UTx in cynomolgus macaques was a major step forward, as there had been no prior reports of successful delivery following UTx in non‐human primates, including baboons. This milestone not only validated the feasibility of UTx in a primate model with close reproductive similarities to humans but also served as a critical foundation for future clinical applications.

The timeline of UTx development in Japan is shown in Figure 1, and various steps have been taken, including the initiation of UTx research in 2009, the first successful live birth following autologous UTx in 2012,^6^ and the first successful live birth following allogeneic UTx in 2020.^7^ In 2018, a request was submitted to the Japan Society of Obstetrics and Gynecology and the Japan Society for Transplantation for the implementation of UTx in Japan. Subsequently, the Japanese Association of Medical Sciences established an ethics review committee to evaluate the procedure. Following approximately 2 years of deliberation, the committee published a report approving the implementation of live donor UTx in Japan. In 2022, an ethical approval application was submitted to the Certified Review Board of Keio University. After a review period of over 2 years, final approval was granted in February 2025, and the clinical development of UTx in Japan is now highly anticipated.

**FIGURE 1:**
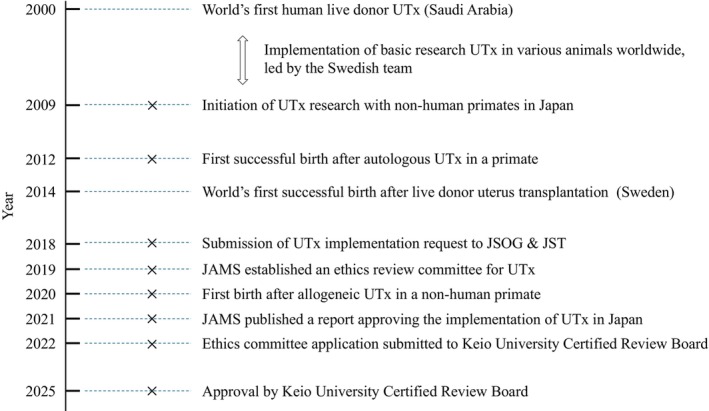
Timeline of uterus transplantation research in Japan. JAMS, the Japanese Association of Medical Sciences; JSOG, the Japan Society of Obstetrics and Gynecology; JST, the Japan Society for Transplantation; UTx, uterus transplantation.

UTx has also expanded across the Asia‐Oceania region, with procedures now performed in China, South Korea, Singapore, and Australia.^8^ It is encouraging to observe that Singapore, where we previously supported cynomolgus macaque UTx experiments, has successfully expanded into human clinical applications.^9^ Brännström et al. emphasized the essential role of systematic preclinical research for ensuring the safety and efficacy of UTx before human trials began. Extensive studies using rodents, pigs, sheep, and non‐human primates have refined surgical techniques, immunosuppressive protocols, and graft preservation strategies. Preclinical research in non‐human primates has played a crucial role in validating the feasibility of UTx, given its close resemblance to human reproductive physiology. These studies have significantly contributed toward establishing the scientific foundation necessary for advancing UTx to clinical application.

UTx represents a paradigm shift in reproductive medicine. Beyond offering women with absolute uterine factor infertility the possibility of biological motherhood, it has driven advancements in transplantation surgery and immunosuppressive management. Further refinements in surgical techniques, optimization of immunosuppressive regimens, and increased accessibility will be crucial for ensuring the long‐term success and sustainability of UTx programs worldwide.

As UTx programs continue to expand globally, it is essential to recognize that the successful implementation of this technology is rooted in decades of rigorous animal research. We extend our highest respect to the Swedish team for their pioneering work and unwavering dedication, which have inspired global advancements in this field. The translation of foundational animal research into clinical practice stands as a testament to the power of sustained scientific inquiry and international collaboration.

## FUNDING INFORMATION

The authors have nothing to report.

## CONFLICT OF INTEREST STATEMENT

The authors declare no conflict of interest.

## Data Availability

The data that support the findings of this study are available from the corresponding author upon reasonable request.
